# Should SGLT2i be used prior to transcatheter edge‐to‐edge repair for secondary mitral regurgitation?

**DOI:** 10.1002/clc.23603

**Published:** 2021-03-27

**Authors:** Neal M. Dixit, Ali Nsair, Marcella A. Calfon Press

**Affiliations:** ^1^ Department of Medicine David Geffen School of Medicine at UCLA Los Angeles California USA; ^2^ Division of Cardiology David Geffen School of Medicine at UCLA Los Angeles California USA

Secondary mitral regurgitation (MR) in patients with heart failure with reduced ejection fraction (HFrEF) portends a poor prognosis when it occurs.^1^ Left ventricular dilation leads to annular dilation of the mitral annulus and reduced coaptation of the mitral valve leaflets leading to secondary MR. Until recently, medical and cardiac resynchronization therapy was the primary treatment strategy for symptomatic secondary MR, with surgical repair reserved for select patients most often undergoing concomitant open heart surgery. The most recent American College of Cardiology/American Heart Association guidelines on the management of valvular heart disease now recommend (Class 2a) mitral valve transcatheter edge‐to‐edge repair (TEER)as a treatment option for many patients who remain symptomatic despite optimal guideline‐directed medical therapy (GDMT). MitraClip® (Abbott), the first and only FDA‐approved percutaneous device to repair MR, has been shown to reduce the regurgitant flow of secondary MR and provide substantial morbidity, mortality and symptomatic benefit (Figure 1). Two major trials of TEER for treatment of secondary MR highlighted the necessity of GDMT optimization prior to intervention.^2^, ^3^ Importantly, these trials have highlighted the role of patient selection in determining which patients benefit the most from mitral valve TEER. For example, patients who are not on GDMT prior to mitral valve TEER benefit less compared to those that remain symptomatic despite maximally tolerated GDMT. As advances in GDMT occur, the role and timing of mitral valve TEER should be continually assessed. Sodium‐glucose cotransporter‐2 inhibitors (SGLT2i) are the newest therapy to show significant benefit in patients with HFrEF.^4^, ^5^ The potential use of SGLT2i prior to mitral valve TEER will be discussed in detail below.

**FIGURE 1:**
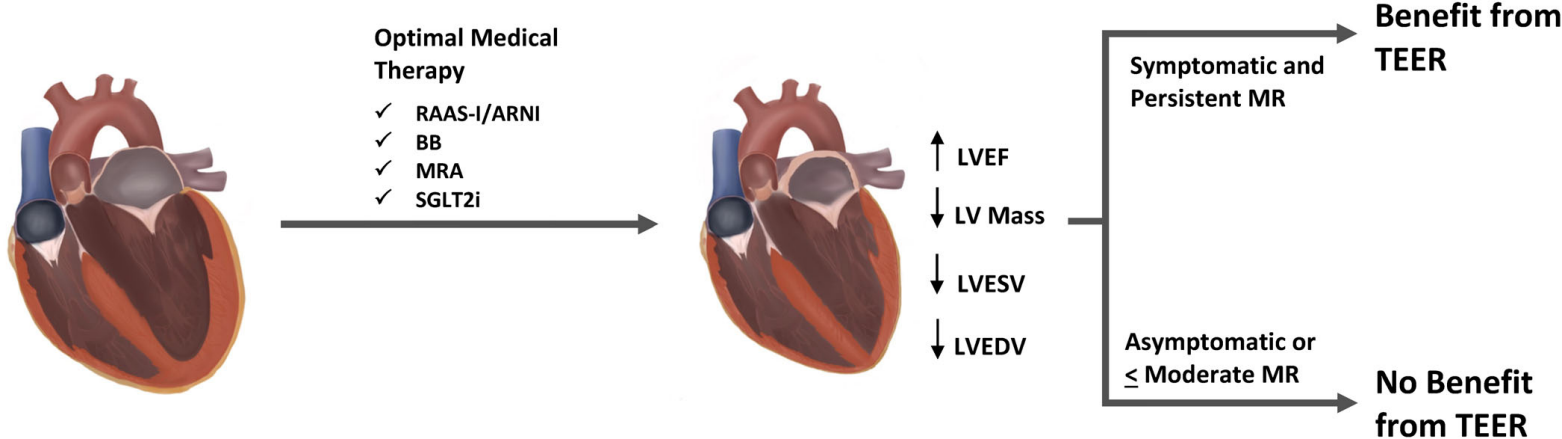
Effect of optimal medical therapy prior to transcatheter mitral valve repair. Optimal medical therapy with agents proven to reverse cardiac remodeling is necessary in order to achieve benefit from mitral valve TEER. Once optimized, only those who remain symptomatic with > moderate MR will benefit from intervention. ARNI, angiotensin receptor‐neprilysin inhibitor; BB, beta‐blocker; LV, left ventricular; LVEDV, left ventricular end diastolic volume; LVEF, left ventricular ejection fraction; LVESV, left ventricular end systolic volume; MR, mitral regurgitation (secondary); MRA, mineralocorticoid receptor antagonist; RAAS‐I, renin‐angiotensin‐aldosterone system inhibitor; SGLT2i, sodium‐glucose cotransporter‐2 inhibitor; TEER, transcatheter edge‐to‐edge‐repair (mitral valve)

Mitral valve TEER for HFrEF patients has been studied in two major trials, COAPT^2^ and MITRA‐FR,^3^ with starkly different results. Published simultaneously in 2018, COAPT showed a 47% reduction in hospitalizations and a 38% reduction in all‐cause mortality among heart failure patients with symptomatic moderate‐to‐severe or severe secondary MR and left ventricular ejection fraction (LVEF) ≤50% despite maximally tolerated GDMT.^2^ However, MITRA‐FR showed no benefit in a similar patient population.^3^ A review of the significant outcome differences by Senni et al identified three likely reasons for better outcomes in the COAPT cohort: (1) a case selection with more severe secondary MR but smaller left ventricular volumes, (2) more successful device implantation and fewer post‐operative complications, and (3) more intensive medical optimization prior to mitral valve TEER.^6^

While both trials required patients to be on maximally tolerated GDMT, COAPT evaluated for this measure under the review of a central committee of experts while MITRA‐FR relied on local center approval.^6^ In the end, GDMT optimization in COAPT was likely superior under the rigorous review of a committee of heart failure specialists. In contrast, the lack of surveillance by a centralized authority in MITRA‐FR was a significant missed opportunity to ensure the best outcome of the intervention.

There is overwhelming evidence that neurohormonal modulation by GDMT leads to reverse cardiac remodeling with resultant reduction in left ventricular size and volume, leading to reduction of MR severity and improvement in functional status.^1^ Renin‐angiotensin‐aldosterone system inhibitors (RAAS‐I), beta‐blockers (BB), and mineralocorticoid receptor antagonists (MRA) have been the mainstays of GDMT for many years, and are proven to reverse cardiac remodeling, promoting improvements in New York Heart Association class and mortality among patients with HFrEF.

Angiotensin receptor‐neprilysin inhibitors (ARNI), Entresto® (Novartis), have been a significantly potent therapy in patients with HFrEF.^7^ Superior to RAAS‐I in the PARADIGM‐HF and PIONEER‐HF trials, ARNI also confer additional benefits of cardiac reverse remodeling (PROVE‐HF) and have become a new foundational therapy in HFrEF.

Another breakthrough in medical therapy have been the SGLT‐2i, which have demonstrated significant benefit in both diabetic and non‐diabetic patients with HFrEF as seen in the seminal DAPA‐HF trial.^4^ Although, SGLT2i were not included as part of optimal medical therapy in the COAPT and MITRA‐FR trials (the two trials preceded the landmark SGLT2i‐HFrEF trials), there is robust evidence that SGTL2i should be initiated in patients with LVEF ≤40% prior to evaluation for mitral valve TEER.

First, SGLT2i provide an impressive reduction in rehospitalization and all‐cause mortality, 25% and 13% in metanalysis, which would potentiate beneficial effects of mitral valve TEER.^4^ Second, SGLT2i promote switching of myocardial fuel from glucose to more efficient ketone bodies, free fatty acids, and branched chain amino acids which reduce maladaptive left ventricular (LV) remodeling.^8^ A recent study highlighted the early and substantial reverse remodeling effects of SGLT2i.^5^ In a group of 84 patients without diabetes, the SGLT2i empagliflozin (compared to placebo) significantly reduced LV end diastolic volume (−25.1 vs. −1.5 ml, *p* < .001), LV end systolic volume (−26.6 vs. −0.5 ml, *p* < .001) and LV mass (−17.8 vs. 4.1 g, *p* < .001). Decreased LV sphericity, increased LVEF (6.0 vs. −0.1, *p* < .001), and increase in 6‐min walk distance (81 vs. −35 m, ∆ from baseline, *p* < .001) were also noted, providing strong evidence for quick LV reverse remodeling and functional benefit occurring within the 6 month study period. MR was not reported but improvement can be inferred from reduction in LV size and a dramatic improvement in symptoms based on the Kansas City Cardiomyopathy Questionnaire (21.0 vs. 1.9 pts, *p* < .001). Finally, the effects of SGLT2i in HFrEF are conveniently achieved at the initial dosage and up‐titration is not required.^4^

In conclusion, the diverging results of COAPT and MITRA‐FR clearly demonstrate TEER for secondary MR was effective after optimal medical therapy for HFrEF as it stood several years ago. Now SGLT2i have emerged as the newest pillar of management in HFrEF, with a likely class effect shown by the DAPA‐HF and EMPEROR‐reduced trials. On the backbone of BB, RAAS‐I/ARNI and MRA, SGLT2i promote significant symptomatic, morbidity, mortality, and LV reverse remodeling benefits. As medical therapy for HFrEF continues to evolve, reserving mitral valve TEER for patients with persistent MR on modern regimens is logical and should be confirmed with future studies.

## CONFLICT OF INTEREST

Dr Marcella A. Calfon Press is a proctor for Mitraclip® (Abbott). Dr Ali Nsair and Dr Neal M. Dixit have nothing to disclose.

## Data Availability

Data sharing not applicable to this article as no datasets were generated or analysed during the current study
